# Aging alters immune responses to vaccines

**DOI:** 10.18632/aging.202598

**Published:** 2021-01-28

**Authors:** Jennifer Connors, Elias K. Haddad, Constantinos Petrovas

**Affiliations:** 1 Department of Microbiology and Immunology, Drexel University College of Medicine, Philadelphia, PA 19320, USA; 2 Department of Medicine, Drexel University College of Medicine, Philadelphia, PA 19320, USA; 3 Institute of Pathology, Department of Laboratory Medicine and Pathology, Lausanne University Hospital, University of Lausanne, Lausanne, Switzerland

**Keywords:** aging, germinal center, vaccines, adjuvants, T-follicular helper cells

Aging is associated with immunosenescence which leads to severe consequences of infection as well as inefficient protection after vaccination. This process is defined by immune remodeling of the innate and adaptive branches of the immune system. One effect of this change is that current vaccine strategies do not sufficiently protect older adults hence the development of efficient approaches could substantially improve morbidity and mortality rates for these under protected people. Aging affects the quality and quantity of antibody responses, including specificity and class of antibody produced. High-affinity antibodies are produced in the germinal centers (GC) of secondary lymphoid tissues where processes like affinity maturation and somatic hypermutation (SHM) take place. The decreased ability of older adults to produce a protective antibody response is due, in part, to defects in T cells. In addition to impairments at the haematopoietic stem cell and lymphoid progenitor level in the bone marrow and thymus, the reduction in clonal diversity of naïve T cells and the skewing of their function to a Th1 pro-inflammatory role [1], likely play an important role in the reduction of immune responsiveness in the elderly. B cell function may suffer from suboptimal follicular CD4 T cell help, but additional intrinsic changes in B cells play a major role in aging antibody production [2]. In human studies, activated B cells show a decline in expression of transcription factors and enzyme activation-induced cytidine deaminase (AID). Due to this decline in AID expression and suboptimal T cell helper function, switch memory B cells, such as IgG+ and IgA+ B cells, experience steep decline in number and frequency with age [3].

Another large factor in the progression to a fulfilled adaptive immunity is the innate immune response. Initiation of the innate response is triggered when pathogen-associated molecular patterns (PAMPS) engage pattern recognition receptors (PRRs) in cells including monocytes, macrophages, epithelial cells, and dendritic cells (DCs). Activation of these key innate cells leads to induction of type I interferon (IFN) responses that mediate anti-viral immunity and initiate and support the adaptive immune system through their functions, such as phagocytosis and antigen presentation. Alterations resulting from aging in these pathways have a crucial role in what is called ‘inflammaging’ or the chronic basal production of pro-inflammatory cytokines like IL-6, TNF-α, and type I IFNs [4]. This deficit, coupled with an altered innate composition including decreased macrophages, monocytes, neutrophils, results in overall decreased vaccine and infection response. Furthermore, circulating pro-inflammatory cells may not reflect accurately the corresponding dynamics in lymphoid organs and particularly in the GC area, which calls for more relevant tissue studies [5]. Key for a productive immune response, DCs, and stromal cells like follicular DCs (FDC) which define the light zone of the GC, support GC reactions to produce high quality antibody by priming T-follicular helper cells (Tfh) in the spleen or lymph nodes (LN), and bind and store immune complexes for presentation to GC B cells [5]. This ensures that only the GC B cells that express B cell receptors (BCRs) with high affinity can compete for antigen. However, human studies have shown defects in cytokine production of DC subsets following stimulation of toll-like receptors (TLR) 4 and 8 which may have consequences for initiation of CD4 T cell differentiation towards Tfh phenotypes. Furthermore, how aging affects human stromal cells including FDCs and their impact in aging GC formation and function is still largely unknown.

**Figure 1:**
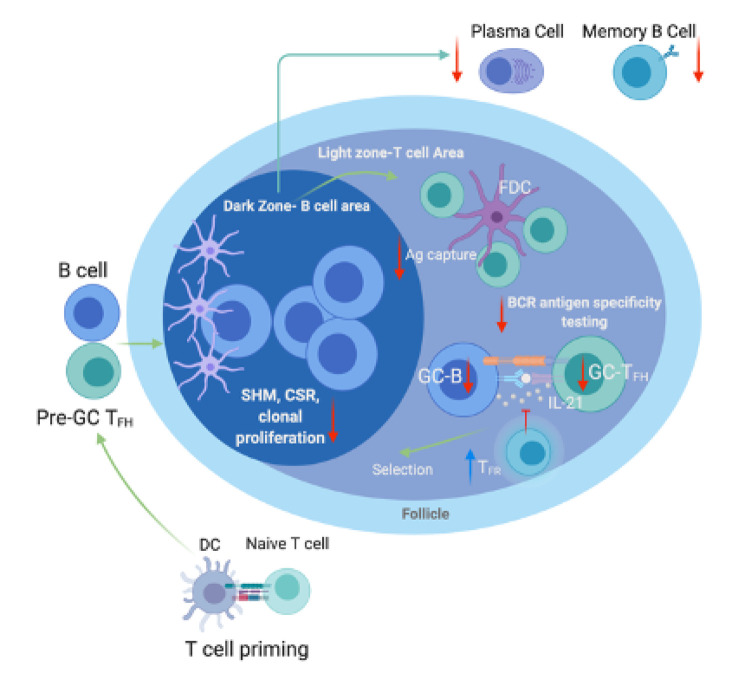
**Age-associated changes in the germinal center of lymph nodes and spleens.** The germinal center (GC) is a specialized microenvironment formed within secondary lymphoid tissues like the lymph nodes (LN) or spleen during infection or immunization. The GC is divided into two compartments: (1) the dark zone where B cells undergo proliferation and somatic hypermutation (SHM) and (2) the light zone where antigen is presented on follicular dendritic cells (FDCs) to B cells that will present to T follicular helper cells (Tfh) for selection. The process is regulated by T follicular regulatory cells (Tfr). After receiving these signals, the B cell will re-enter the dark zone to undergo further proliferation and SHM and will exit as memory B cells or high-affinity antibody secreting plasma cells. In aging, these processes are dysregulated. Not only is the microarchitecture of the spleen and LN physically restructured, it has been shown that there are fewer Tfh and proliferating GC-B cells while the number of follicular monocytes, granulocytes, and Tfr are increased resulting in a more suppressive repertoire with fewer class-switched plasma and memory B cells. This dysregulation partly explains why we see decreased infection and vaccination responses in older adults. *Figure created with Biorender.com*

GCs, through virtue of their function, are critical for potent immune responses against foreign antigen. The production of high-affinity antibody results from complex interactions of DCs and naïve T cells, followed by B and Tfh cells in the GC reaction. Any deviation from this complex dance, results in aberrant humoral immunity as we see in aging individuals in response to vaccine or infection. Along with restructuring of the microarchitecture of the spleen and lymph nodes, GCs from older nonhuman primates (NHP) have recently been shown to have altered baseline cell composition including decreased follicular Tfh, proliferating GC B cells and follicular monocytes, granulocytes, and increased T-follicular regulatory (Tfr) cells [6,7] (Figure 1). Understanding and targeting these specific changes could be valuable in adapting new adjuvant therapy in vaccines that are less effective in older adults including influenza and SARS-CoV-2 vaccines. These high-specificity adjuvants would target a declining cell population or signaling pathway within a cell population that will result in a boost of vaccine efficacy. Additionally, since the Tfr/Tfh ratio is skewed toward Tfr, targeting this cell type to block this highly suppressive function could increase Tfh cells and therefore, memory B cell and long-lived plasma cells response after infection or vaccination. It might also be beneficial to look at adjuvant therapy that will target DCs for improved pre-Tfh priming or antigen capture and presentation by FDCs in GC responses. Recently, we have reported that a novel adjuvant, adenosine deaminase (ADA-1), enhances the ability of Tfh to provide B cell help by creating a pro-Tfh microenvironment and, when administered as a molecular adjuvant in a HIV DNA vaccine, improves GC Tfh and B cell phenotype in the spleens and LN of vaccinated mice. This increase is also associated with increased env-binding antibody in the serum [8]. Better understanding of the interplay that controls GC responses in aging will undeniably have clinical applications and will be required to address the rising issue of the elderly and vaccination. We invite the scientific community to discuss the clinical benefit of this promising new research adjuvant and applications.
